# In Vitro Culture of *Aegle marmelos* Against Media Composition Stress: Molecular Identification, Media, and Enzyme Optimization for Higher Growth Yields

**DOI:** 10.1155/ijog/4630425

**Published:** 2025-04-14

**Authors:** Magdy I. Bahnasy, Ashraf B. Abdel Razik, Mohamed F. Ahmed, Mohamed A. Nasser, Getachew Tekle Mekiso, Eman Z. Ahmed, Eman T. Hussien

**Affiliations:** ^1^Forestry and Timber Tree Research Department, Horticulture Research Institute, Agriculture Research Center, Giza, Egypt; ^2^Genetic Department, Faculty of Agriculture, Ain Shams University, Cairo, Egypt; ^3^Dry and Saline Farming Technology Department, Arid Land Agricultural Graduate Studies and Research Institute, Ain shams University, Cairo, Egypt; ^4^Department of Horticulture, Faculty of Agriculture, Ain Shams University, Cairo, Egypt; ^5^Department of Statistics, Wachemo University, Hosaina, Ethiopia; ^6^Botany and Microbiology Department, Faculty of Science, Helwan University, Cairo, Egypt

**Keywords:** *Aegle marmelos*, catalase, ISSR, micropropagation, morphological and physiological description, Murashige and Skoog medium, woody plant media

## Abstract

*Aegle marmelos*, known for its spiky appearance, is a versatile tree found worldwide. In the Indian medical tradition, this therapeutic tree is utilized to treat various ailments. It is commonly propagated through seeds, although they have a limited lifespan and are susceptible to insect damage. Due to the variability of seed offspring, standardized varieties are not readily available. Molecular identification was performed for the plant species to be as a fingerprint identification based on genomic basic. Hence, this study manipulated the in vitro multiplication for enhancing *Aegle marmelos* traits through variation in media type and composition. In phase one of the experiment, successful micropropagation has been easily achieved with shoot tip culture on two growth in vitro media: Murashige and Skoog (MS) medium and woody plant medium (WPM) with different concentrations (one-fourth, one-half, three-fourths, and full power media) with two sucrose concentration 20 and 30 g/L. The growth parameters measured indicated a heightened response to both MS and WPM media, each with its distinct composition. The genetic variation via intersimple sequence repeat (ISSR) molecular marker in the first phase was 35.5%. In phase two, the hormonal treatment was applied for the best media choice from Phase 1. During the second phase of multiplication and rooting stages with phytohormones, the optimal treatments were chosen to maximize yields. In the multiplication stage, the most favorable conditions, as determined by morphological parameters, were achieved with full MS medium supplemented with 30 g sucrose, 0.1 mg/L Kin, and 0.75 mg/L BAP. In contrast, for the rooting stage, the optimal treatment consisted of one-fourth MS medium supplemented with 15 g sucrose, 0.5 mg/L Kin, 0.1 g/L activated charcoal, and 15 mg/L IBA. Physiological parameters exhibited variability, with each metabolite displaying distinct optimal conditions. Catalase plays a crucial role in decomposing hydrogen peroxide to protect cells, tissues, and organs. This research effectively enhanced the in vitro micropropagation of *Aegle marmelos* by determining the most efficacious medium formulations and hormonal treatments for shoot multiplication and roots, while also illustrating the influence of WPM on catalase enzyme activity enhancement.

## 1. Introduction

The bael (*Aegle marmelos* L. Correa), a member of the Rutaceae family, is a highly valued tree species in South Asia, known for its economic significance [1]. In India, it is also known as begal-quince, golden apple, and stone apple, and holds sacred importance in Hindu-majority areas. Throughout history, Indians and other South Asian inhabitants have revered bael for its culinary and medicinal uses in both Ayurvedic and folk medicine systems [2]. Due to its fragrance and versatility, every part of the bael tree possesses significant medicinal properties. Leaves, fruits, bark, roots, and seeds are all utilized in treating various ailments including dysentery, dyspepsia, neurological diseases, and rheumatism. Beyond its medicinal applications, bael is utilized in industrial food processing and pharmaceutical extraction, highlighting its multifaceted economic importance [3–5]. Bael extracts have shown antibacterial and antifungal [6] and antiviral activities [7]. An antidiarrheal activity reported by Shoba and Thomas [8] could also be due to the same or similar compounds.

The leaves and twigs of *A. marmelos* trees serve as fodder, while its wood, characterized by a potent aroma upon cutting, boasts versatile applications. Despite its hard, gray–white nature, the wood is prone to warping and cracking during curing and is not notably durable. Nonetheless, it finds utility in a range of endeavors including carts, construction, carving, turnery, tool and knife handles, pestles and combs, and polishing. This tree, featuring a thick trunk, is typically found in dry forests and exhibits resilience in enduring prolonged droughts [9].

Similar to primary medical care, medicinal plants play a vital role in the lives of underprivileged populations worldwide. Traditional therapies, which often involve plant extracts, are utilized by approximately 80% of the world's nations. Bael, an Indian medicinal plant, has been traditionally employed to treat various illnesses and has yielded numerous bioactive chemicals. Extracts and essential oils are obtained from Bael plants, and their phytoconstituents are characterized. To extract the most active compounds, selective solvents and standard operating procedures are employed on the preferred plant parts, including roots, fruits, leaves, flowers, or stems [10, 11].

The main vegetative method of bael propagation is through root suckers, which is a labor-intensive and sluggish procedure. In the meantime, seeds are not a viable option for replication due to their short shelf life and susceptibility to insect damage. Additionally, commercial propagation using grafting and cutting methods is not possible. However, successful regeneration of the important medicinal plant *Aegle marmelos* has been achieved using various explants such as epicotyl, cotyledon, hypocotyl, and root explants. These explants were sourced from axenic seedlings of *A. marmelos* aged 4 weeks and cultured on Murashige and Skoog (MS) medium supplemented with plant growth regulators [12–16].

To use bael as a cash crop and significantly advance the rural economies of many nations, tissue culture protocols for efficient multiplication, biochemical characterization for essential phytochemicals, and genetic characterization of the resulted individual plants are required [5]. It is unlikely that bael will undergo extensive genetic characterization using DNA markers. The intersimple sequence repeat (ISSR) marker, MP2-based DNA fingerprinting, and the randomly amplified polymorphic DNA (RAPD) marker, OPA2, were used to confirm the tissue-cultured bael plants' true to typeness. For bael germplasm, the use of molecular marker-based DNA fingerprinting is essential to comprehend domestication events, as well as to calculate appropriate population sizes for conservation, accurately identify the best cultivars for extensive clonal propagation, and map genes and quantitative trait loci (QTL) [17, 18].

Catalase enzymes are found in all living organisms, spanning from unicellular prokaryotes to multicellular eukaryotes. The emergence of catalytic enzymes can be traced back to around 3.5 billion years ago, coinciding with the initiation of aerobic respiration in primitive planktonic bacteria. Subsequently, the evolution of catalase continued alongside the formation of the aerobic biosphere on Earth. All aerobic organisms engage in both photosynthesis and respiration within their cells, resulting in the production of detrimental reactive oxygen species (ROS), specifically superoxide anion (O_2_^−^), hydroxyl radical (OH), hydrogen peroxide (H_2_O_2_), and singlet oxygen (^1^O_2_) [19, 20].

This study is aimed at increasing the nutritional and antioxidant value of *Aegle marmelos* fruit via changing the in vitro culture media type and composition. Also, an estimate of the genetic variation could be obtained as a result for these different culture conditions. Decisions must be made regarding whether to use only local germplasm for the agronomically significant traits or whether to introduce germplasm from the region of origin, India, to have much greater genetic diversity for better success in research and development, once the overall picture of the bael germplasm has been revealed. By determining the best medium compositions and hormonal treatments for shoot multiplication and rooting, this study effectively enhanced the in vitro micropropagation of *Aegle marmelos*.

## 2. Materials and Methods

### 2.1. Plant Materials

Homogenous seeds of one tree of *Aegle marmelos* were collected from an animal Zoo in Giza in September 2021. The seeds were identified based on data described in Boulos [21]. The coordinates of the seed location were as follows: latitude 30°1⁣′45.12⁣^″^ N, longitude 31°12⁣′47.16⁣^″^ E.

### 2.2. Initiation of *Aegle marmelos* In Vitro Culture

About 20 seeds were sterilized in 40% Clorox for 30 min and then washed with sterile distilled water three times for 5 min/time. After that, the sterilized seeds were transferred into free MS media. The seeds germinated after 21 days and then subcultured three times on free MS for 4 weeks to harden the plant.

### 2.3. Molecular Identification

The molecular identification of beal plants was performed as a fingerprint for this plant species. The whole genomic DNA of the plantlet was isolated according to Deshmukh et al. [22] and estimated on 1% agarose gel. The 18SrRNA technique was applied using the specific primers: EUK1. f, 5⁣′-AGCGGAGGAAAAGAAACTA – ⁣′3; and EUK2. r, 5⁣′-TACTAGAAGGTTCGATTAGTC – ⁣′3. PCR reaction mixture consists of 12.5 *μ*L of the master mix, 1 *μ*L of each primer, and 50 ng DNA template. The PCR program began with an initial denaturation step, lasting 5 min at 95°C. The PCR reaction continued with 35 cycles of 30 s at 94°C, 30 s at the annealing temperature of 54°C, and 1 min extension at 72°C.The final extension was at 72°C for 10 min for one cycle. The purified 18S rRNA gene amplicons were analyzed directly by sequencing, using an ABI 3730xl DNA sequencer by using forward and reverse sequences, by combining the traditional Sanger with the new 454 technology (GATC Company, Germany) according to Saqib et al. [23]. The blast was identified, and the phylogenetic tree was designed by MEGA7 software.

### 2.4. Phase 1

#### 2.4.1. Experimental Design

In this stage, the explant materials (shoot tips) were exposed to three variable parameters (in 10 replicates): media type, media concentration, and sucrose concertation. The explants were cultured on two different types of in vitro culture media (MS and WPM), different media concentrations (one-fourth, one-half, three-fourths, and full power) with different concentrations of sucrose (20 and 30 g/L) for each media type (Table 1). The nodal segments were incubated for 5 weeks at 25 ± 2°C for 16 h light/8 h dark. The entire germplasm of bael was surveyed and characterized for variations in all possible morphological and physiological characteristics with estimation for the genetic variation using the ISSR molecular marker.

#### 2.4.2. Morphological Traits

The impact of various cultural conditions on *A. marmelos* is evident in its morphological traits. To gauge this variation, parameters such as fresh weight, shoot length, and leaf number were assessed. Due to the limited size of the produced plantlet, additional parameters for measurement are not feasible.

#### 2.4.3. Physiological Parameters

Changes in the growth media are reflected in the plant's internal responses and metabolites. Three replicates of each physiological parameter were estimated, including total pigmentation, total soluble carbohydrates, and total protein, were measured to assess these alterations. The estimation of variable metabolites was conducted in the following manner: the total pigmentation was determined using the methodology outlined by Metzener et al. [24]; the total soluble sugars were determined using the methodology outlined by Umbriet et al. [25]; and the total proteins were determined using the methodology specified by Bradford [26]. The methods in question have undergone recent modifications as documented by Hussien et al. [27] and Ahmed et al. [28].

#### 2.4.4. Genetic Fidelity Testing

The genetic variation resulted from the variable treatments of media compositions of *A. marmelos* in the first phase was estimated by the ISSR molecular marker. The total reaction consists of 12.5 *μ*L PCR master mix, 2 *μ*L of ISSR primers, and 2 *μ*L of DNA sample and is completed with dH_2_O up to the final volume of 25 *μ*L. The PCR tube was set in the thermocycler machine (Bio-Rad) with the following program: 95°C for 5 min followed by 35 cycles of 95°C for 30 min, 54°C for 30 min, and 72°C for 1 min. Six primers were applied, and only three of them gave reproducible bands. The final product was checked on 1.8% agarose gel electrophoresis and analyzed via Bio-Rad Quantity One software to detect band scoring and measure the genetic polymorphism and variation.

### 2.5. Phase 2

This phase was represented in the multiplication and rooting stages. The best media constituents were selected from the establishment stage based on the best growth parameters measured. After that, apply different growth phytohormones to get the best yield for this fruit, at two stages: multiplication and rooting.

#### 2.5.1. Experimental Design

Based on the results of the first stage, both full MS with 30 g sucrose and three-fourths WPM with 20 g sucrose were the best free media composition for growth parameters. For the multiplication stage, both these two media were examined with variable phytohormone concentrations as follows: 0.1 mg/L Kin with (0.5, 0.75, and 1 mg/L) BAP. For the rooting stage, both one-fourth MS free media were examined with 15 g sucrose and one-fourth WPM free media with 15 g sucrose. The activated charcoal was applied to activate rooting with a concentration of 0.1 g/L. Both these two media types were adjusted with the following phytohormones: 0.5 mg/L Kin with (5, 10, 15, and 20 mg/L) IBA. Ten replicates were performed for this experiment. The nodal segments were incubated for 5 weeks at 25 ± 2°C for 16 h light/8 h dark. The variable multiplication and rooting treatments are illustrated in Table 2.

#### 2.5.2. Morphological and Physiological Parameters

In both multiplication and rooting stages, due to the hormonal additions, the morphological parameters were more obvious, and more parameters were measured from three replicates like the following: plantlet fresh weight, shoot length, number of branches, and number of leaves, where the physiological parameters estimated were as follows: total pigmentation, total proteins, total carbohydrates, total phenolics, and total flavonoids. The total phenolics were estimated according to the protocol of Kujala et al. [29], whereas the total flavonoids were estimated according to Zhishen et al. [30]. All these protocols were recently modified according to Tawfik and Abdel Razik [31]. The catalase enzyme activity was measured following the protocol of Góth [32].

### 2.6. Statistical Analysis

The analysis involved the examination of gel electrophoresis pictures, wherein bands that were detected were assigned a value of 1, while bands that were absent were assigned a score of 0. Jaccard's coefficient of similarity was utilized to generate a pairwise similarity matrix, while the unweighted pair group method with the arithmetic averaging algorithm (UPGMA) was employed to conduct cluster analysis and generate a dendrogram. The calculations were performed using Bio-Rad Quantity One (4.6.2) [33].

Descriptive analysis of the variance test and one-way ANOVA were conducted on the collected data using Minitab 19. The mean average was computed, along with correlations and standard deviations. ANOVA was employed to determine if there were any statistically significant disparities among the means of each of the parameters being examined. The link between all Populus treatments was evaluated using the Community Analysis Package (CAP) (1.2) software, based on morphological, physiological, and molecular data. The CAP methods used were complete linkage clustering and PCA blot for PCA covariance ordination.

## 3. Results

### 3.1. Molecular Identification

**Figure 1:**
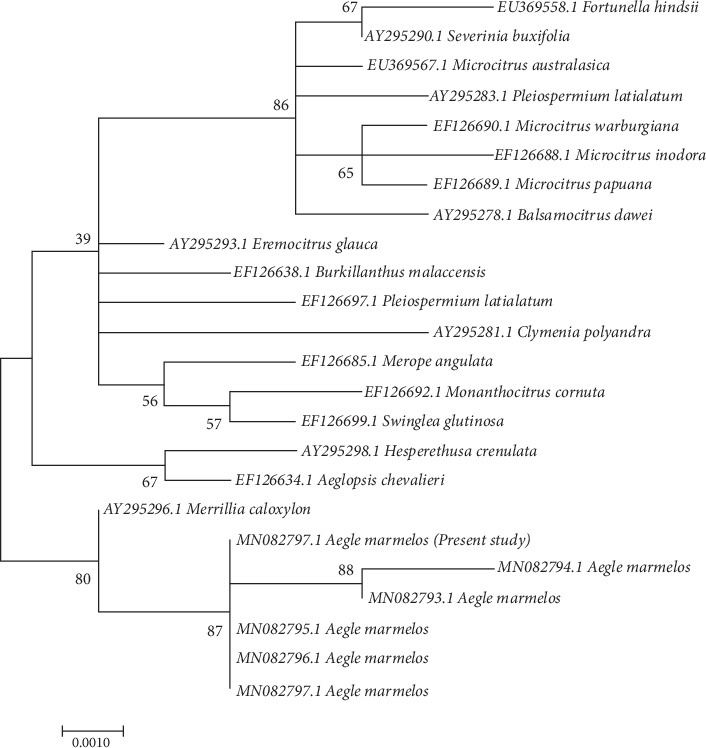
Molecular phylogenetic analysis by maximum likelihood method based on the Jukes–Cantor model. The tree with the highest log likelihood (−1695.41) is shown. The percentage of trees in which the associated taxa clustered together is shown next to the branches. Initial trees for the heuristic search were obtained by applying the neighbor-joining method to a matrix of pairwise distances estimated using the maximum composite likelihood (MCL) approach. The tree is drawn to scale, with branch lengths measured in the number of substitutions per site. The analysis involved 24 nucleotide sequences. All positions containing gaps and missing data were eliminated. Evolutionary analyses were conducted in MEGA7.

Following DNA extraction, the species was determined at the molecular level using the 18S rRNA-PCR technique and eukaryote-specific primers. To identify the plant species, the product was sequenced, and the resulting sequence was aligned with NCBI data; then, the data was exported to MEGA7 software (Figure 1).

The evolutionary relationships of *Aegle marmelos* with other species within the Rutaceae family and potentially other related families are presumably depicted in the phylogenetic tree. It can determine the species that are closest to it and offer valuable information on the divergence and speciation processes that have taken place throughout evolution. The utilization of phylogenetic analysis serves to validate the taxonomic positioning of *Aegle marmelos* within the botanical family. Researchers can ascertain the position of a species within the Rutaceae family and its relationship to other genera and species within the family by comparing its DNA sequences with those of other species.

### 3.2. Phase 1: Establishment Stage

#### 3.2.1. In Vitro Conditions and Morphological and Physiological Parameters

**Figure 2:**
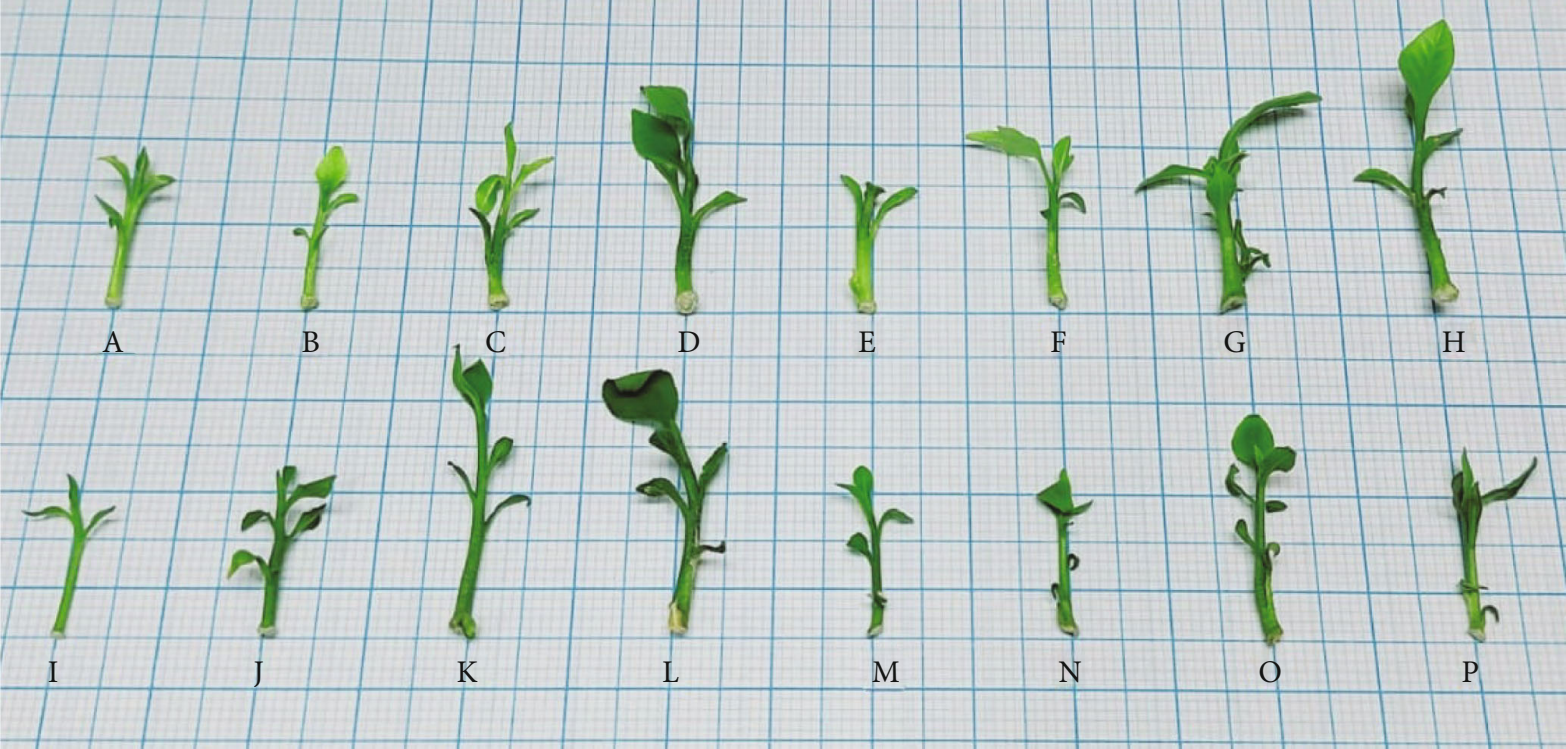
The in vitro cultured plantlets of *Aegle marmelos* resulted from variable media types and compositions. The codes are illustrated in Table 1.

The in vitro conditions of *Aegle marmelos* culture in different media types (MS and WPM) with different media strengths (one-fourth, one-half, three-fourths, and full strength) resulted in variable 16 treatments as shown in Figure 2. It is obvious that the growth after 4 weeks is limited.

In response to the variable treatment, the morphological traits showed variable behavior (Table 3 and Figure 2). For shoot length, the best treatments were D, L, O, and P (according to Table 1), which is most with full power media, either MS or WPM. For leaf number, the best treatments were as follows: H and O (according to Table 1). For fresh weight, the best treatments were as follows: D, L, and C (codes according to Table 1), which means that either full MS or full WPM enhances the fresh weight.

Based on the morphological results, there are positive correlations among fresh weight and shoot length, fresh weight and leaf number, and shoot length and leaf number. The same treatments enhance all these parameters (Table 4).

In Table 5, there is a complete illustration for the effect of media types on measured physiological parameters. The types and compositions of culture media significantly influence the physiological characteristics of *Aegle marmelos*. Treatment D exhibits the highest pigment content (1.3246 ± 0.0045), while Treatment H displays the lowest (0.2530 ± 0.0026). This indicates that specific culture media formulations, such as those utilized in Treatment D, enhance pigment formation. Treatment I exhibits the highest soluble sugar level at 146.31 ± 0.357, greatly surpassing the other treatments. The minimal values are documented in Treatments F and G (56.320 ± 0.439 and 56.343 ± 0.433, respectively). This suggests that Treatment I creates advantageous conditions for sugar buildup, but Treatments F and G may be inadequate. Treatment A (90.513 ± 0.446) exhibits the highest total protein content, while Treatment B (29.567 ± 0.422) demonstrates the lowest. This indicates that Treatment A includes elements that markedly improve protein synthesis. The studies indicate that the choice of culture media substantially influences physiological responses. Additional examinations of the constituents of the most efficacious treatments (e.g., D, I, and A) may enhance growth conditions for *A. marmelos* in tissue culture research.

Based on physiological parameters, there is a positive correlation between total soluble sugars and total protein. There is a negative correlation between total pigments with both total proteins and total soluble sugars (Table 6).

**Figure 3:**
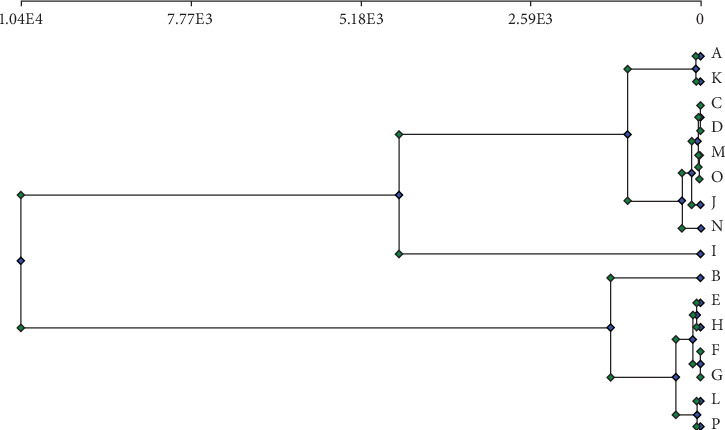
Total dendrogram of *Aegle marmelos* in response to the variable culture media types and compositions based on morphological and physiological parameters (codes are in Table 1).

**Figure 4:**
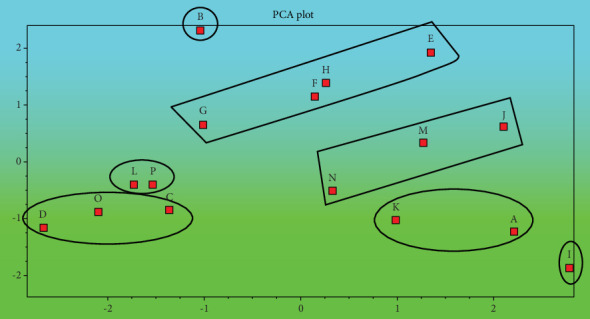
PCA plot of *Aegle marmelos* in response to the variable culture media types and compositions based on morphological and physiological parameters (codes are in Table 1).

The dendrogram in Figure 3 and the PCA plot in Figure 4 illustrated the grouping of the variable treatments of *Aegle marmelos* resulted from variable media types and compositions. The grouping indicated that most of the treatments with MS media were closer to each other than treatments with WPM growth media.

**Figure 5:**
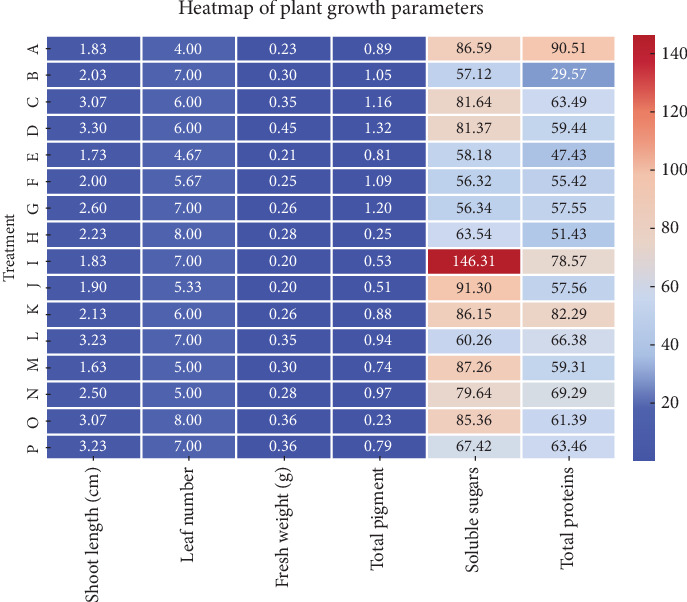
Heat map analysis based on plant growth parameters based on morphology and physiology.

The heat map (in Figure 5) offers a distinct visual depiction of the fluctuations in plant development characteristics among various treatments. The largest concentration of soluble sugars is found in Treatment I (146.31 mg/g), markedly exceeding the other treatments. This may signify a robust reaction to that treatment. Treatments A, K, and I exhibit elevated total protein levels, indicating a potential favorable impact on protein accretion. Likewise, shoot length is maximized in treatments C, D, L, O, and P, suggesting possible growth-enhancing effects. Treatment D (1.3246 mg/g) exhibits the highest total pigment levels, which may be linked to elevated chlorophyll or carotenoid accumulation. The fresh weight values exhibit a narrower range compared to other parameters, indicating that treatments exert a lesser influence on biomass accumulation than on biochemical parameters such as soluble sugars and pigments. Treatment H exhibits a significantly low total pigment level (0.253 mg/g), rendering it an outlier in comparison to other treatments. This may suggest stress or inadequate photosynthetic efficiency.

#### 3.2.2. Genetic Variation

**Figure 6:**
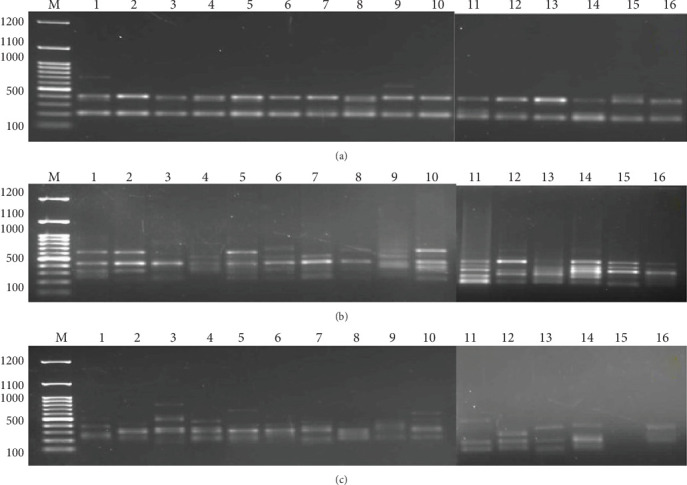
Gel banding pattern of ISSR molecular marker of *Aegle marmelos* in response to the variable culture media types and compositions.

The banding patterns generated by the ISSR marker were compared to determine the genetic polymorphism among the treatments of plantlets. The primers selected because they had good amplification compared to the others were UBC811, UBC818, and UBC849 (Table 7). The gel banding pattern is demonstrated in Figure 6. The total polymorphism percentage resulted from amplification with these primers was 35.5%.

### 3.3. Phase 2 (Multiplication and Rooting Stages)

#### 3.3.1. Morphological and Physiological Parameters

**Figure 7:**
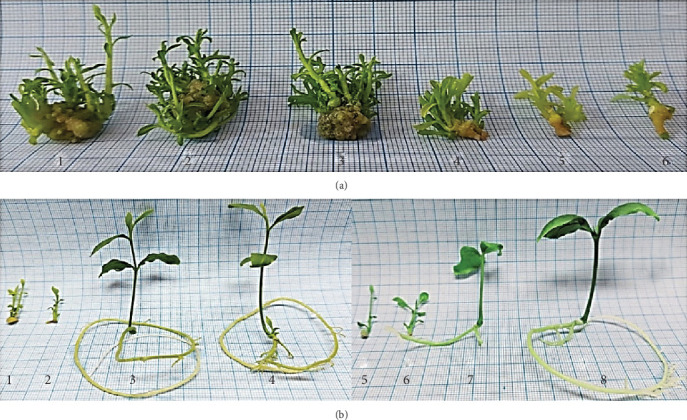
The morphological behavior of in vitro culture of *Aegle marmelos*: (a) multiplication stage and (b) rooting stage (codes are in Table 2).

In this phase, the in vitro plantlets were exposed to phytohormones in the multiplication stage beside activated charcoal in the rooting stage to enhance the growth parameters of *Aegle marmelos*. For the multiplication stage, the best conditions, according to the morphological parameters, were full MS with 30 g sucrose, 0.1 mg/L Kin, and 0.75 mg/L BAP; while for the rooting stage, the best treatment was one-fourth MS with 15 g sucrose, 0.5 mg/L Kin, 0.1 g/L activated charcoal, and 15 mg/L IBA. The physiological parameters varied, and each metabolite has different best conditions. The morphological description of the multiplication stage is illustrated in Table 8, whereas the rooting stage is in Table 9, both grouped in Figure 7. The physiological parameters of the multiplication stage are illustrated in Table 10, whereas the rooting stage is in Table 11.

**Figure 8:**
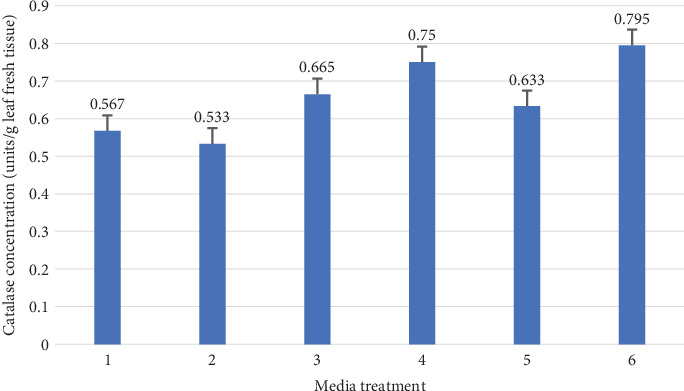
The activity of catalase enzyme in response to the media composition in the multiplication stage of *Aegle marmelos* (codes illustrated in Table 2 multiplication stage).

The catalase enzyme activity is clearly having a role in resistance to environmental stress; as it dissociates H_2_O_2_ into water and oxygen in an energy-efficient manner in the stressed cells. The catalase enzyme activity was estimated in best chosen treatments in the second phase of the experiment. It was clear that treatments of WPM media with 0.5 and 1 mg/L cytokinin were the best treatments to have the highest catalase activity, with values of 0.75 and 0.759 *μ*M, respectively (Figure 8).

## 4. Discussion

The current study is based on the selection of the best conditions to obtain the highest growth yield of in vitro *Aegle marmelos* culture. These conditions included media type, salt strength, sucrose concentration, and variable phytohormones. The work is separated into two phases: The first one is the establishment stage to select the best media type, solute strength, and sucrose concentration and then followed by the second phase of in vitro multiplication and rotting stages with the treatment of variable phytohormones (Kin and BAP). The selection of the best media constituents depends on morphological and physiological growth parameters. The ISSR molecular marker was applied to estimate the genetic variation reflected in the somaclonal variation.

The in vitro cultivation of *Aegle marmelos* is essential in plant biotechnology, particularly in enhancing growth across diverse media formulations. The composition of the media stressfully impacts the growth parameters of Aegle marmelos, influencing shoot length, leaf count, biomass accumulation, and biochemical characteristics including total pigments, soluble sugars, and proteins. Optimizing media conditions is crucial for improving biomass production and biochemical yield. Research indicates that differences in macro and micronutrient composition might influence plant metabolism and enzyme functions, consequently affecting growth rates and stress responses. Molecular identification methods, including DNA barcoding and RAPD analysis, are essential for confirming genetic stability in in vitro cultures and evaluating stress-induced changes [34]. Optimizing enzymes, especially in stress-tolerant cultures, boosts antioxidant enzyme activity, which helps alleviate oxidative stress and enhances overall plant resilience [35]. Consequently, an optimally designed media composition, along with molecular and enzymatic assessments, guarantees enhanced growth yields and consistent regeneration of Aegle marmelos in controlled in vitro environments.

The application of tissue culture technique to control the growth of the plants agreed with Tawfik and Abdel Razik [31] who proved that tissue culture-based micropropagation is frequently praised as an effective method for enhancing forest tree crops. Elite mother plants may be chosen, propagated in large numbers using tissue culture, and expanded. Sidana et al. [36] explained the tissue culture requirements for the shooting of bael. Arya and Shekhawat [37] tissue cultured elite bael trees' auxiliary and terminal buds using explants that were 8–10 mm in size [38].

In this study, the application of phytohormones aligns with Surana [38], who suggested harvesting juvenile buds during the vegetative stage to enhance success rates. Additionally, combining 100 mg/L ascorbic acid and 150 mg/L citric acid was recommended to prevent browning, corroborating previous findings. The induction of callus was accomplished using the MS medium containing 5 mg/L, 2-4D, and naphthalene acetic acid (NAA). To induce rooting, kinetin (KIN) (1 mg/L) and BAP (0.5 mg/L) were used. The use of cotyledonary nodes of bael allowed for high-frequency plantlet regeneration. The precise requirements for plant growth regulators for effective bael shooting and rooting, as well as the quantity of workers who tried using various explants, were described by Nayak et al. [39]. Through enhanced auxiliary branching of the mother plant, in vitro clonal propagation of bael was optimized, and DNA fingerprinting was used to confirm the true to typeness of the daughter plants [18].

Cotyledonary nodes of *Aegle marmelos* experienced high-frequency plantlet regeneration. Cotyledonary nodes from *A. marmelos* seedlings that had been in vitro grown for 1 month were cultured on MS medium that had been supplemented with benzyl adenine (BA), KIN, and indole-3-acetic acid (IAA), either separately or in combination. With an average shoot number of 487.5 per explant in a 7-week period, the medium containing 6.6 *μ*M BA + 1.14 *μ*M IAAshowed the greatest regenerative response in 86.6% of the cultures. Cultures kept on a KIN-supplemented medium responded extremely poorly. Auxins IAA, indole-3-butyric acid (IBA), or NAA was added to a half-strength MS root induction medium before being transferred to in vitro responded shoots. In medium supplemented with 14.7 M IBA, rooting performed best. With an 80% survival rate, rooted plantlets were acclimated and released into the field [40]. The application of the ISSR molecular marker was supported by Princy et al. [41] who proved that using nodal segments, a successful protocol has been created for the quick clonal propagation of *Aegle marmelos*. To verify the genetic fidelity of these regenerated plantlets, an ISSR marker assay was used. The variation in the genomic content of beal plants could be further reflected in the physiological metabolic pathways which are then expressed morphologically in variable behavior and growth parameters.

The activity of catalase is considered a significant predictor of the antioxidant activity of plants due to its ability to withstand drought and soil salinity. H_2_O_2_ is present in response to various stressful conditions, including heat shock, metallic stress, pathogenic infections in plants, and photooxidative processes induced by abiotic stressors such as cold, drought, salinity, and ozone stress [42].

## 5. Conclusions

Indigenous plants, specifically the bael, have a substantial impact on mitigating concealed hunger. Due to its abundant food, medicinal, and other beneficial properties, this tree species has great potential for large-scale cultivation.

The primary objective of this study was to achieve the maximum yield of *Aegle marmelos* in vitro growth, with a specific emphasis on identifying optimal growing conditions. The parameters encompassed in this study comprised the media type, salt content, sucrose concentration, and the variability of phytohormones. The task consists of two distinct stages. The initial stage, known as the establishment stage, involves the selection of optimal media type, solute strength, and sucrose content. Subsequently, the second phase of in vitro multiplication and rotting stages commenced with the utilization of various phytohormones, namely, KIN, IBA, and BAP. It is imperative to select the most suitable media components by considering both morphological and physiological growth factors. The optimal in vitro conditions for multiplication, as determined by the findings of Phase 2, were MS with 30 g sucrose, 0.1 mg/L Kin, and 0.75 mg/L BAP. Similarly, during the rooting stage, the most effective treatment was ^1^ MS with 15 g sucrose, 0.5 g/L Kin, 0.1 g/L activated charcoal, and 15 mg/L IBA. The ISSR molecular marker was utilized to quantify the genetic variation that is manifested in the somaclonal variation. H_2_O_2_, a nonradical ROS, serves as the substrate for the catalase enzyme. The enzyme under consideration is responsible for the metabolic process of neutralization through the breakdown of H_2_O_2_, so assuring the maintenance of an adequate cellular concentration of the molecule. Additionally, this molecule plays a vital role in other cellular signaling interactions. From the variable treatments, it was clear that WPM with phytohormone (cytokinin) enhances the catalase enzyme activity more than plantlets treated on MS media.

## Figures and Tables

**Table 1:** The codes for variable treatments in media composition of *Aegle marmelos* in the establishment stage.

**Code**	**MS treatment**	**Code**	**WPM treatment**
A	1/4 MS + 30 g sucrose	I	1/4 WPM + 30 g sucrose
B	1/2 MS + 30 g sucrose	J	1/2 WPM + 30 g sucrose
C	3/4 MS + 30 g sucrose	K	3/4 WPM + 30 g sucrose
D	Full MS + 30 g sucrose	L	Full WPM + 30 g sucrose
E	1/4 MS + 20 g sucrose	M	1/4 WPM + 20 g sucrose
F	1/2 MS + 20 g sucrose	N	1/2 WPM + 20 g sucrose
G	3/4 MS + 20 g sucrose	O	3/4 WPM + 20 g sucrose
H	Full MS + 20 g sucrose	P	Full WPM + 20 g sucrose

**Table 2:** The codes for variable treatments in media composition of *Aegle marmelos* in multiplication and rooting stage with phytohormones.

**Multiplication stage conditions**						
**Code**	**Media type**	**Media strength (g/L)**	**Sucrose (g/L)**	**Activated charcoal (g/L)**	**Cytokinin Kin (mg/L)**	**Cytokinin BAP (mg/L)**

1	MS	Full	30	0	0.1	0.5
2	MS	Full	30	0	0.1	0.75
3	MS	Full	30	0	0.1	1
4	WPM	3/4	20	0	0.1	0.5
5	WPM	3/4	20	0	0.1	0.75
6	WPM	3/4	20	0	0.1	1

**Rooting stage conditions**						
**Code**	**Media type**	**Media strength (g/L)**	**Sucrose (g/L)**	**Activated charcoal (g/L)**	**Cytokinin Kin (mg/L)**	**Cytokinin IBA (mg/L)**

1	MS	1/4	15	0.1	0.5	5
2	MS	1/4	15	0.1	0.5	10
3	MS	1/4	15	0.1	0.5	15
4	MS	1/4	15	0.1	0.5	20
5	WPM	1/4	15	0.1	0.5	5
6	WPM	1/4	15	0.1	0.5	10
7	WPM	1/4	15	0.1	0.5	15
8	WPM	1/4	15	0.1	0.5	20

**Table 3:** Morphological means of *Aegle marmelos* in response to the variable culture media types and compositions (mean ± SD).

**Treatment**	**Shoot length (cm)**	**Leaf number**	**Fresh weight (g)**
A	1.833 ± 0.057^efg^	4.00 ± 0.000^f^	0.230 ± 0.000^fg^
B	2.033 ± 0.057^cde^	7.00 ± 0.000^b^	0.303 ± 0.005^c^
C	3.066 ± 0.057^a^	6.00 ± 0.000^c^	0.350 ± 0.000^b^
D	3.300 ± 0.000^a^	6.00 ± 0.000^c^	0.446 ± 0.005^a^
E	1.733 ± 0.115^fg^	4.667 ± 0.577^e^	0.206 ± 0.005^h^
F	2.000 ± 0.000^cde^	5.667 ± 0.577^df^	0.250 ± 0.000^ef^
G	2.600 ± 0.000^b^	7.00 ± 0.000^b^	0.263 ± 0.011^de^
H	2.233 ± 0.057^c^	8.00 ± 0.000^a^	0.283 ± 0.005^cd^
I	1.833 ± 0.115^efg^	7.00 ± 0.000^b^	0.203 ± 0.005^h^
J	1.900 ± 0.000^def^	5.333 ± 0.577^cde^	0.203 ± 0.005^gh^
K	2.133 ± 0.115^cd^	6.00 ± 0.000^c^	0.263 ± 0.011^de^
L	3.233 ± 0.115^a^	7.00 ± 0.000^b^	0.350 ± 0.000^b^
M	1.633 ± 0.115^g^	5.00 ± 0.000^de^	0.300 ± 0.000^c^
N	2.500 ± 0.000^b^	5.00 ± 0.000^de^	0.283 ± 0.011^cd^
O	3.066 ± 0.057^a^	8.00 ± 0.000^a^	0.363 ± 0.011^b^
P	3.233 ± 0.115^a^	7.00 ± 0.000^b^	0.360 ± 0.000^b^
*F*-value	180.59⁣^∗∗∗^	66.84⁣^∗∗∗^	295.90⁣^∗∗∗^

*Note:* Superscripts resulted from the statistical analysis of Tukey's analysis of ANOVA. “a” is an indication for the highest value, and as the number increases, the value decreases.

⁣^∗∗∗^Highly significant.

**Table 4:** Correlation among the morphological parameters in the establishment stage.

	**Shoot length**	**Leaf number**
Leaf number	0.472	—
Fresh weight	0.844	0.365

**Table 5:** Physiological means of *Aegle marmelos* in response to the variable culture media types and compositions (mean ± SD).

**Treatment**	**Total pigment**	**Soluble sugars**	**Total proteins**
A	0.8896 ± 0.0025^i^	86.593 ± 0.514^cd^	90.513 ± 0.446^a^
B	1.0533 ± 0.0020^f^	57.117 ± 0.312^kl^	29.567 ± 0.422^m^
C	1.1626 ± 0.0030^d^	81.637 ± 0.456^f^	63.490 ± 0.463^f^
D	1.3246 ± 0.0045^a^	81.367 ± 0.437^f^	59.443 ± 0.497^h^
E	0.8050 ± 0.0040^j^	58.180 ± 0.157^k^	47.430 ± 0.475^l^
F	1.0936 ± 0.0030^e^	56.320 ± 0.439^l^	55.420 ± 0.465^j^
G	1.2033 ± 0.0035^c^	56.343 ± 0.433^l^	57.550 ± 0.412^i^
H	0.2530 ± 0.0026^o^	63.540 ± 0.375^i^	51.433 ± 0.464^k^
I	0.5256 ± 0.0041^m^	146.31 ± 0.357^a^	78.567 ± 0.389^c^
J	0.5056 ± 0.0035^n^	91.297 ± 0.265^b^	57.557 ± 0.411^i^
K	0.8833 ± 0.0041^i^	86.153 ± 0.193^de^	82.293 ± 0.140^b^
L	0.9450 ± 0.0030^h^	60.263 ± 0.234^j^	66.380 ± 0.385^e^
M	0.7450 ± 0.0036^l^	87.257 ± 0.207^c^	59.310 ± 0.105^h^
N	0.9736 ± 0.0025^g^	79.643 ± 0.273^g^	69.287 ± 0.313^d^
O	1.2273 ± 0.0020^b^	85.363 ± 0.405^e^	61.387 ± 0.474^g^
P	0.7943 ± 0.0030^k^	67.423 ± 0.362^h^	63.463 ± 0.460^f^
*F*-value	23462.68⁣^∗∗∗^	12036.15⁣^∗∗∗^	3615.37⁣^∗∗∗^

*Note:* Superscripts resulted from the statistical analysis of Tukey's analysis of ANOVA. “a” is an indication for the highest value, and as the number increases, the value decreases.

⁣^∗∗∗^Highly significant.

**Table 6:** Correlation among the physiological parameters in the establishment stage.

	**Total pigment**	**Soluble sugars**
Soluble sugars	−0.305	—
Total proteins	−0.070	0.568

**Table 7:** ISSR primers code, nucleotide sequence, and variation of *Aegle marmelos* in response to the variable culture media types and compositions.

**No**	**Name**	**Seq (5**⁣′**-3**⁣′**)**	**Total bands**	**Total polymorphic bands**	**Polymorphism percentage (%)**
1	UBC811	GAG AGA GAG AGA GAG AC	3	1	33.33
2	UBC818	CAC ACA CAC ACA CAC AG	6	2	33.33
3	UBC849	GAGAGAGAGAGAGAGAT	5	2	40
Total			14	5	35.5

**Table 8:** Morphological means of *Aegle marmelos* in response to the variable culture media types and compositions with phytohormones in the multiplication stage (mean ± SD).

**Treatment**	**Shoot length (cm)**	**Shoot number**	**Leaf number**	**Fresh weight (g)**
1	3.833 ± 0.033^a^	16.00 ± 0.000^b^	122.00 ± 0.000^b^	0.980 ± 0.000^b^
2	3.400 ± 0.000^b^	25.00 ± 0.000^a^	172.00 ± 1.000^a^	1.080 ± 0.000^a^
3	3.133 ± 0.033^c^	14.00 ± 0.000^c^	112.33 ± 0.333^c^	0.960 ± 0.000^c^
4	2.233 ± 0.033^d^	9.000 ± 0.000^d^	53.000 ± 0.000^d^	0.260 ± 0.000^d^
5	2.000 ± 0.000^e^	5.000 ± 0.000^e^	30.667 ± 0.333^e^	0.140 ± 0.000^e^
6	2.000 ± 0.000^d^	2.000 ± 0.000^f^	19.000 ± 0.000^f^	0.116 ± 0.003^f^
*F*-value	1029.60⁣^∗∗∗^	---	17689.47⁣^∗∗∗^	114970.60⁣^∗∗∗^

*Note:* Superscripts resulted from the statistical analysis of Tukey's analysis of ANOVA. “a” is an indication for the highest value, and as the number increases, the value decreases. ---: nonsignificant.

⁣^∗∗∗^Highly significant.

**Table 9:** Morphological means of *Aegle marmelos* in response to the variable culture media types and compositions with phytohormones in the rooting stage (mean ± SD).

**Treatment**	**Shoot length (cm)**	**Shoot number**	**Leaf number**	**Fresh weight (g)**	**Root length (cm)**	**Root number**
1	2.90 ± 0.000^e^	2.00 ± 0.000^b^	11.6 ± 0.333^a^	0.11 ± 0.000^e^	0.00 ± 0.000^f^	0.00 ± 0.000^b^
2	2.26 ± 0.033^h^	1.00 ± 0.000^h^	9.00 ± 0.000^b^	0.05 ± 0.000^g^	0.50 ± 0.000^e^	1.00 ± 0.000^a^
3	8.26 ± 0.033^b^	1.00 ± 0.000^g^	6.00 ± 0.000^d^	0.65 ± 0.003^b^	31.5 ± 0.000^a^	1.00 ± 0.000^a^
4	9.16 ± 0.033^a^	2.00 ± 0.000^a^	9.00 ± 0.000^b^	0.61 ± 0.000^c^	22.6 ± 0.033^b^	1.00 ± 0.000^a^
5	2.70 ± 0.000^f^	1.00 ± 0.000^f^	5.00 ± 0.000^e^	0.05 ± 0.000^g^	0.00 ± 0.000^f^	0.00 ± 0.000^b^
6	2.43 ± 0.033^g^	1.00 ± 0.000^e^	7.00 ± 0.000^c^	0.07 ± 0.000^f^	0.60 ± 0.000^e^	1.00 ± 0.000^a^
7	5.60 ± 0.000^d^	1.00 ± 0.000^d^	4.00 ± 0.000^f^	0.28 ± 0.000^d^	7.26 ± 0.033^d^	1.00 ± 0.000^a^
8	7.10 ± 0.000^c^	1.00 ± 0.000^c^	4.00 ± 0.000^f^	0.66 ± 0.000^a^	15.6 ± 0.033^c^	1.00 ± 0.000^a^
*F*-value	14538.25⁣^∗∗∗^	---	542.71⁣^∗∗∗^	58019.29⁣^∗∗∗^	356307.81⁣^∗∗∗^	---

*Note:* Superscripts resulted from the statistical analysis of Tukey's analysis of ANOVA. “a” is an indication for the highest value, and as the number increases, the value decreases. ---: nonsignificant.

⁣^∗∗∗^Highly significant.

**Table 10:** Physiological means of *Aegle marmelos* in response to the variable culture media types and compositions with phytohormones in the multiplication stage.

**Treatment**	**Total phenolics**	**Flavonoids**	**Total pigments**	**Carbohydrates**	**Protein**
1	52.36 ± 0.556^e^	4.717 ± 0.625^c^	0.24 ± 0.010^a^	36.86 ± 0.799^e^	137.3 ± 0.551^c^
2	87.59 ± 0.525^c^	14.46 ± 0.503^a^	0.21 ± 0.010^bc^	46.68 ± 0.595^d^	110.0 ± 1.000^e^
3	50.59 ± 0.525^f^	5.373 ± 0.546^c^	0.12 ± 0.010^d^	45.63 ± 0.548^d^	90.53 ± 0.503^f^
4	93.46 ± 0.503^b^	12.76 ± 0.681^b^	0.22 ± 0.010^ab^	61.61 ± 0.537^c^	158.5 ± 0.451^b^
5	84.59 ± 0.525^d^	11.56 ± 0.513^b^	0.13 ± 0.010^d^	87.78 ± 0.705^a^	128.4 ± 0.529^d^
6	95.19 ± 0.756^a^	12.63 ± 0.551^b^	0.19 ± 0.010^c^	66.86 ± 0.804^b^	176.6 ± 0.529^a^
*F*-value	3810.10⁣^∗∗∗^	156.84⁣^∗∗∗^	72.90⁣^∗∗∗^	2253.26⁣^∗∗∗^	7640.06⁣^∗∗∗^

*Note:* Superscripts resulted from the statistical analysis of Tukey's analysis of ANOVA. “a” is an indication for the highest value, and as the number increases, the value decreases.

⁣^∗∗∗^Highly significant.

**Table 11:** Physiological means of *Aegle marmelos* in response to the variable culture media types and compositions with phytohormones in the rooting stage (mean ± SD).

**Treatment**	**Total phenolics**	**Flavonoids**	**Total pigments**	**Carbohydrates**	**Protein**
1	52.86 ± 0.799^bc^	6.630 ± 0.548^bc^	0.383 ± 0.005^b^	91.48 ± 0.501^d^	137.4 ± 0.513^a^
2	47.69 ± 0.602^c^	5.630 ± 0.548^c^	0.470 ± 0.010^a^	116.5 ± 0.500^c^	110.5 ± 0.500^c^
3	50.43 ± 0.512^c^	5.563 ± 0.512^c^	0.340 ± 0.010^c^	87.38 ± 0.541^e^	120.5 ± 0.500^b^
4	49.35 ± 0.563^c^	6.617 ± 0.539^bc^	0.496 ± 0.015^a^	51.51 ± 0.500^g^	120.8 ± 1.040^b^
5	57.65 ± 0.569^ab^	5.577 ± 0.517^c^	0.416 ± 0.020^b^	66.88 ± 0.827^f^	87.86 ± 0.808^e^
6	59.64 ± 0.558^a^	8.617 ± 0.539^a^	0.476 ± 0.015^a^	140.4 ± 0.513^b^	75.56 ± 0.513^g^
7	59.41 ± 0.522^a^	7.653 ± 0.566^ab^	0.386 ± 0.025^b^	139.5 ± 0.500^b^	100.5 ± 0.513^d^
8	51.41 ± 5.570^c^	7.347 ± 0.566^ab^	0.330 ± 0.010^c^	162.9 ± 0.921^a^	81.40 ± 0.529^f^
*F*-value	15.88⁣^∗∗∗^	12.69⁣^∗∗∗^	51.62⁣^∗∗∗^	11884.94⁣^∗∗∗^	3427.93⁣^∗∗∗^

*Note:* Superscripts resulted from the statistical analysis of Tukey's analysis of ANOVA. “a” is an indication for the highest value, and as the number increases, the value decreases.

⁣^∗∗∗^Highly significant.

## Data Availability

All data of this work are available here.
