# Supplementary figures and images for: In Vitro Culture of Aegle marmelos Against Media Composition Stress: Molecular Identification, Media, and Enzyme Optimization for Higher Growth Yields
Source: Int J Genomics. 2025 Apr 14;2025:4630425. doi: 10.1155/ijog/4630425 (PMC12011463; doi:10.1155/ijog/4630425)

**Supplementary material**

**Graphical Abstract**


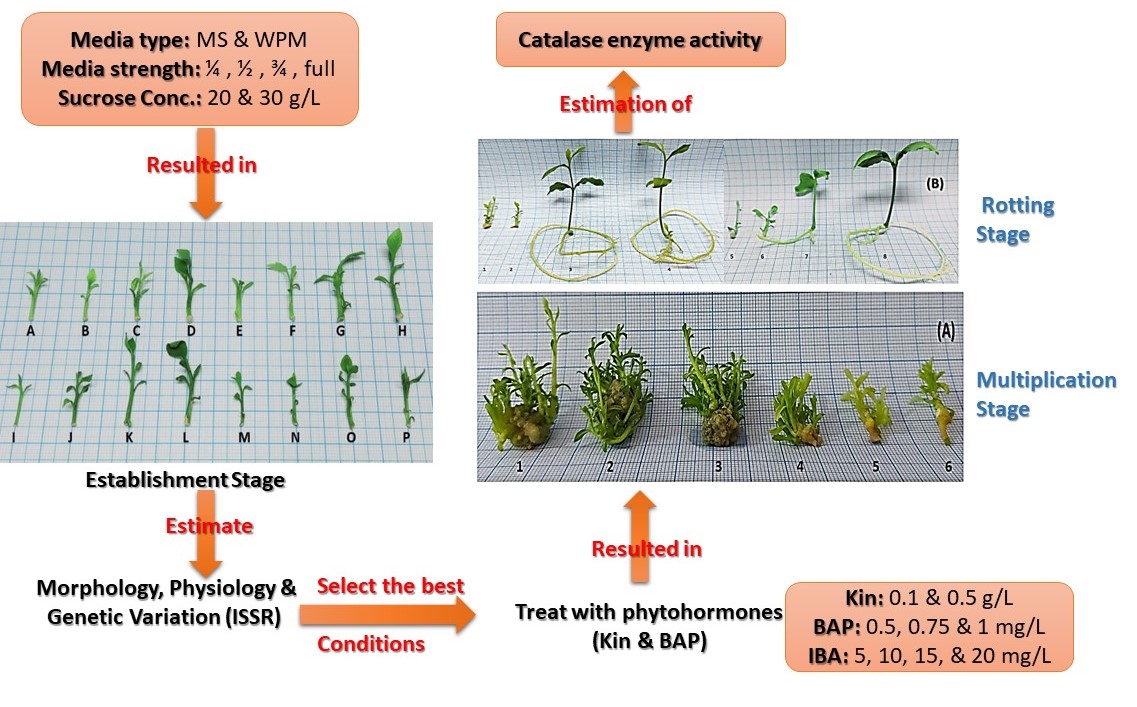

Supplement: Supporting Information — Additional supporting information can be found online in the Supporting Information section. The graphical abstract was provided as supporting information. [file 4630425.f1.docx]
